# A Comparison of Auditory Perception in Hearing-Impaired and Normal-Hearing Listeners: An Auditory Scene Analysis Study

**DOI:** 10.5812/ircmj.9477

**Published:** 2013-11-05

**Authors:** Arash Bayat, Mohammad Farhadi, Akram Pourbakht, Hamed Sadjedi, Hesam Emamdjomeh, Mohammad Kamali, Golshan Mirmomeni

**Affiliations:** 1 Department of Audiology, Iran University of Medical Sciences, Tehran, IR Iran; 2 Department of Audiology, Ahvaz Jundishapur University of Medical Sciences, Ahvaz, IR Iran; 3 Department and Research Center of Otolaryngology, Head and Neck Surgery, Hazrat Rasoul Akram Hospital, Iran University of Medical Sciences, Tehran, IR Iran; 4 Rehabilitation Research Center, Iran University of Medical Sciences, Tehran, IR Iran; 5 Department of Electronics, Engineering faculty, Shahed University, Tehran, IR Iran; 6 Department of Rehabilitation Management, Iran University of Medical Sciences, Tehran, IR Iran

**Keywords:** Auditory Scene Analysis, Sensorineural Hearing Loss, Hearing

## Abstract

**Background:**

Auditory scene analysis (ASA) is the process by which the auditory system separates individual sounds in natural-world situations. ASA is a key function of auditory system, and contributes to speech discrimination in noisy backgrounds. It is known that sensorineural hearing loss (SNHL) detrimentally affects auditory function in complex environments, but relatively few studies have focused on the influence of SNHL on higher level processes which are likely involved in auditory perception in different situations.

**Objectives:**

The purpose of the current study was to compare the auditory system ability of normally hearing and SNHL subjects using the ASA examination.

**Materials and Methods:**

A total of 40 right-handed adults (age range: 18 - 45 years) participated in this study. The listeners were divided equally into control and mild to moderate SNHL groups. ASA ability was measured using an ABA-ABA sequence. The frequency of the "A" was kept constant at 500, 1000, 2000 or 4000 Hz, while the frequency of the "B" was set at 3 to 80 percent above the" A" tone. For ASA threshold detection, the frequency of the B stimulus was decreased until listeners reported that they could no longer hear two separate sounds.

**Results:**

The ASA performance was significantly better for controls than the SNHL group; these differences were more obvious at higher frequencies. We found no significant differences between ASA ability as a function of tone durations in both groups.

**Conclusions:**

The present study indicated that SNHL may cause a reduction in perceptual separation of the incoming acoustic information to form accurate representations of our acoustic world.

## 1. Background

In everyday life situations, we experience a complex acoustical environment caused by several simultaneously active sound sources which often overlap at any given time (1, 2). Because the acoustic information entering one’s ears is a mixture of all the sounds in the environment, a key function of the auditory system is to determine which acoustic cues in the mixture originate from which sound source and to construct, from the concurrent inputs, neural representations of the sound events that maintain the integrity of the original sources (1-3).

Auditory scene analysis (ASA) refers to the ability of the human auditory system to segregate sounds generated by different acoustical sources in different perceptual streams, and to combine sounds arose from the same acoustical source in a single perceptual stream (3-5). The voice of a single person among a crowd, or the sound of a piano among those of other musical instruments in a symphonic orchestra, provide everyday examples of auditory streams. It has been shown that ASA ability plays an important role in the acquisition of normal speech and language patterns. Indeed, it is believed that auditory scene analysis is the basis of hearing (2, 5). Deficits in ASA may partly underlie hearing difficulties encountered with aging, such as impaired understanding of speech in noisy environments (6, 7), and may contribute also to deficits characteristic of some specific language impairments (8). In laboratory situations, the ASA phenomenon can be demonstrated using sounds with very simple spectral and temporal characteristics, namely, sequences of pure tones which alternate between two frequencies (A and B) in a repeating ABA-ABA pattern, where A and B denote tones of different frequencies, and the hyphen (-) represents a silent gap. It has been shown that these simple stimulus sequences can evoke two dramatically different percepts, depending on spectral and temporal stimulus characteristics (5, 9). When the tones are close in frequency, most listeners report hearing a single stream of tones with an alternating pitch; this percept is called the “stream integration”. In contrast, when the tones are more widely spaced in frequency, the stimulus sequence “splits” perceptually into two streams, as if produced by two separate sound sources; this is called the “stream segregation” (2, 10). Thus, below the stream integration or “fission boundary” (FB) threshold, only a single stream is perceived; and beyond the stream integration or “coherence boundary” (CB) threshold, two distinct streams are heard (i.e. A-A-A and B-B-B).

Micheyl et al. (11) found that thresholds for the discrimination of changes in the frequency of the last B tone in an ABA-ABA sequence were influenced by stimulus parameters known to control the stream segregation of tone sequences. Specifically, they found that thresholds increased (i.e. worsened) as the frequency separation between the A and B tones (ΔF AB) decreased, and that they decreased (i.e. improved) as the tone-presentation rate and overall length of the sequence increased. It seems that streaming involves fairly high level processes in the brain, there is evidence that it takes time to build (a few seconds), and is somewhat dependent upon attention.

It is known that sensorineural hearing loss (SNHL) is associated with deficits in spectro-temporal processing, and could detrimentally impacts understanding speech in challenging listening environments (6, 12). Although loss of audibility is a primary contributor to perceptual deficits experienced by individuals with SNHL, it does not solely account for the perceptual deficits experienced by these patients (6).

It has been shown that even when sounds are amplified (e.g. through hearing aids) so as to restore audibility, the difficulty usually remains (6). These difficulties might arise from poorer ability to perceive and recall auditory sequences (i.e. ASA capacity) in listeners with SNHL than subjects with normal hearing ability.

## 2. Objectives

The purpose of the current study was to compare the auditory system ability of normally hearing and SNHL subjects using the ASA examination.

## 3. Materials and Methods

### 3.1. Subjects

A total of 40 right-handed adults (age range: 18 - 45 years) participated in this investigation. They were recruited from the Audiology Clinic of the Department of Otolaryngology at the Hazrat Rasoul Akram Hospital, Iran University of Medical Sciences. The experimental protocol and all procedures involving human subjects in this study were approved by the ethics committee of Iran University of Medical Sciences. Signed consent forms were obtained from all participants before starting the experiment. The subjects were classified into control and SNHL groups as follows:

#### 3.1.1. Control Group

Control group (n = 20): They all had pure-tone audiometric thresholds of 15 dB HL or better at octave frequencies from 250 to 8000 Hz in both ears.

#### 3.1.2. Sensorineural Hearing Loss

Sensorineural hearing loss (SNHL) group (n = 20): This group had bilateral symmetrical mild to moderate SNHL with pure-tone averages (500, 1000, 2000 Hz) ranging from 34 to 51 dB HL, and monosyllabic word recognition scores of approximately 76% or greater. 3.2. Auditory Scene Analysis (ASA) Assessment

**Figure 1.**
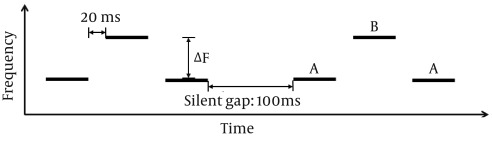
Schematic Representation of Stimuli Used to Study the Auditory Scene Analysis (TD: Tone duration; ΔF: Frequency difference between the A and B tones).

The stimuli were a sequence of "ABA–ABA" tones, where" A" and "B" represent different tone frequencies, and "–" denotes a silent interval (Figure 1). Tone durations (TDs) were set at 50, 100 and 150 ms including 10 ms cosine shaped onset and offset ramps. There was a 20-ms silence between tones, and there was a 100-ms pause (silent interval) after one ABA group. 

Each sequence consisted of five precursor tones at each frequency (i.e. five A tones and five B tones), followed by two target tones (i.e. one A tone and one B tone). For each trial, the frequency of the "A" tone was kept constant at 500, 1000, 2000 or 4000 Hz. The frequency of the "B" tone was set at 3 to 80 percent above that of the" A" tone. In this sequence, the subject usually perceives a single stream (coherence) ABA–ABA when the frequency ratio of A and B is small. Alternatively, when the frequency ratio is large enough, two separate streams are usually heard (fission): "A–A–", and "B–B–".

In this paper, we focused on the fission boundary (FB) threshold because it tends to be more stable, both within and across subjects, than the temporal coherence (TC) threshold (6). FB Thresholds were measured using a two-alternative forced-choice (2AFC) procedure which tracked the 79.4%-correct point on the psychometric function. Tones were 40 dB above the absolute threshold, i.e. at 40 dB SL and were presented using TDH 39 headphones.

### 3.3. Statistical Analysis

Statistical analyses consisted mainly of repeated measures analyses of variance (RM-ANOVAs). Reported P values and degrees of freedom included the Greenhouse–Geisser correction wherever required (i.e. whenever the sphericity assumption was not met). All statistical analyses were performed with SPSS Version 16 (SPSS Inc.).

## 4. Results

**Figure 2.**
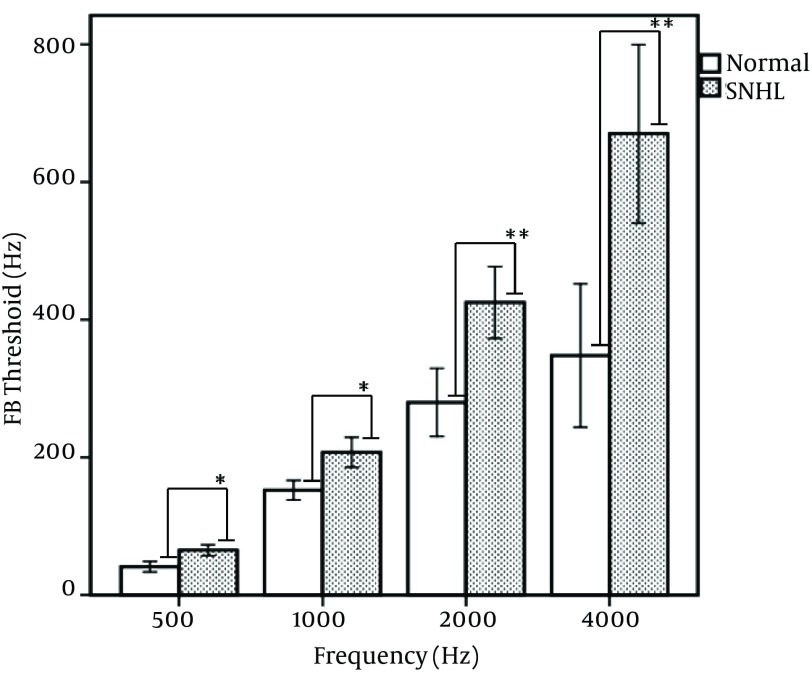
The Mean Values of Fission Boundary Thresholds for Normally Hearing and Sensorineural Hearing Loss (SNHL) Subjects as a Function of Frequency. Error bars Indicate ± One Standard Error. (*: P < 0.05, P < 0.01)

Figure 2 depicts the mean results for both subject groups in the auditory scene analysis test. As is evident from the figure, the mean FB threshold was significantly larger for SNHL group than controls. Additionally, this difference in ASA performance between SNHL and control subjects occurred mainly at higher frequencies. We found no significant differences between FB thresholds as a function of tone durations (TD) in both groups. Therefore, the main difference in FB threshold was primarily based on the frequency separation (ΔF) of the tones rather than TD. The interaction between TD and ΔF variables in both groups -across different frequencies- was not meaningful (P > 0.05). Our findings revealed a significant correlation between the degree of hearing loss and the mean FB threshold, which was stronger in 2000 Hz (P < 0.001, r = 0.71), and 4000 Hz (P < 0.001, r = 0.79). 

## 5. Discussion

Auditory scene analysis (ASA) has been described as the process by which the auditory system separates or groups the individual sounds in natural-world situations. When listening to sequences of sounds, sounds may be grouped together, and so perceived as emanating from a single source (fusion or integration), or perceived as separate auditory streams originating from distinct sources (fission or stream segregation). In the present study, we compared the performance of SNHL and normally hearing adults in an ASA task using alternating high- and low-frequency tones. Our evidences demonstrated that SNHL subjects perceived the sound sequences as segregating into two streams poorer than the control subjects. Our findings are consistent with Mackersie’s findings. Mackersie (13) examined the frequency separation for pure tones at the fission boundary (FB), and determined that hearing-impaired ears require a greater frequency separation than normal hearing ears at this boundary, though there were a few exceptions.

SNHL is mainly characterized by deficits related to the loss of outer hair cell function in the cochlea. It may reduce absolute sensitivity of the auditory system, which leads to detection thresholds larger than normal. Additionally, SNHL results in auditory filters, characterizing the frequency selectivity of the auditory system, to be broader. Thus, the frequency selectivity of the auditory system may be diminished in these patients (14). This result can be explained by the degree of overlap of excitation patterns in the cochlea in response to an acoustic stimulus, with less overlap leading to greater stream segregation (14). Beauvois and Meddis (15) believed that two stimuli must excite separate neural populations to form two separate auditory streams. It seems that the broader auditory filters, of SNHL individuals are responsible for their reduced ability to perform stream segregation (6). Therefore, the deteriorated performance by SNHL listeners especially in high frequency is likely caused by poorer frequency discrimination in these frequency regions. We did not find any statistically significant differences between ASA performances as a function of tone duration (TD) in both groups. Thus, it would be expected that impairments of ASA in SNHL group were not attributable to this parameter. The correlation between pure-tone stream segregation and degree of hearing loss in SNHL patients was significant, so that ASA ability in moderate SNHL subjects was worse than that of mild hearing loss individuals. In fact these losses in peripheral sensitivity in SNHL patients may lead to changes in the neural mechanisms critical for the perceptual separation of the incoming acoustic information, and eventually affect signals being delivered to the central auditory nervous system.

In conclusion, the present study indicated that SNHL may reduce perceptual separation of the incoming acoustic information to form accurate representations of our acoustic world. This impairment is more dependent on spectral aspect of the sound, rather than its temporal component.
